# Is the Public Adequately Informed about #BBL? A Content Analysis of Instagram Posts Regarding the Brazilian Butt Lift Procedure

**DOI:** 10.1007/s00266-025-05019-z

**Published:** 2025-07-02

**Authors:** Iulianna C. Taritsa, Jenna R. Stoehr, Dana W. Shuaibi, Emily S. Chwa, Karlee C. Knight, Hillary Lai, Elizabeth Tran, Arun K. Gosain

**Affiliations:** Division of Plastic Surgery, Ann & Robert H. Lurie Children’s Hospital, Northwestern University Feinberg School of Medicine, 225 E. Chicago Ave., Box 93, Chicago, IL 60611 USA

**Keywords:** Gluteal fat transfer, Body contouring, Safety, Patient education, Social media

## Abstract

**Background:**

Patients often get information on the Brazilian butt lift (BBL) through social media. It is not known how well social media informs patients of the risks associated with the BBL procedure. The objective of this study was to analyze social media content about BBL.

**Methods:**

Instagram was queried using seven relevant hashtags twice in one month. The first 50 relevant posts for each hashtag in the “Top” search category were analyzed for author qualifications, social media engagement, and content, including types of photographs, references to research, educational information, and discussion of safety risks.

**Results:**

A total of 587 posts, with a total of 458,659 “likes” and 9,230 comments, were included in the analysis. The majority (79.0%) of authors were physicians or physician groups. Of 128 unique physicians, 63.0% were board-certified plastic surgeons. Most posts (73.0%) showed postoperative results, and 39.0% were overt advertisements. While 29.0% of posts included some educational information, only 12.0% mentioned risks and only 3.0% referenced research. Board-certified plastic surgeons contributed 57.0% of the educational posts. Only 33.0% of the posts mentioned risk. Board-certified plastic surgeons were more likely to produce content related to research, but were less likely to discuss safety risks than non-board-certified physicians.

**Conclusions:**

Most BBL-related content on Instagram does not provide educational information to patients or promote understanding of the risks of the procedure. Board-certified plastic surgeons should consider using social media to disseminate information on the risks and indications of BBL and other popular cosmetic procedures.

**Level of Evidence IV:**

This journal requires that authors assign a level of evidence to each article. For a full description of these Evidence-Based Medicine ratings, please refer to the Table of Contents or the online Instructions to Authors www.springer.com/00266.

## Introduction

The Brazilian butt lift (BBL) is an increasingly popular cosmetic procedure in recent years [1]. Unlike similar cosmetic procedures designated as “lifts” (i.e., facelift, breast lift), a BBL does not remove excess skin or reposition sagging skin [2]. Rather, a BBL is an augmentation of the gluteal region which begins with liposuction followed by fat transfer into the gluteal region [3]. As a result of the procedure, the gluteal region will appear fuller and more symmetric, and areas of the body where fat was removed will have a smoother shape [2]. Despite its increasing popularity, the BBL is considered to be one of the highest risk esthetic procedures, largely due to the risk of intravascular fat injection, resulting in fat embolism and death [4].

Several studies have been reported on the techniques by which plastic surgeons can avoid serious complications while performing a BBL [1,5–7]. The authors advise surgeons to keep gluteal fat injection superficial in the subcutaneous tissue, in contrast to intramuscular injection. The gluteal muscles are considered to be “danger zones” where underlying gluteal vessels are present and where fat grafting is more likely to enter the vasculature [1,8]. It is also recommended that cannulas larger than 4 mm in diameter be used, as they are more likely to follow the intended path and not bend, avoiding inadvertent injection in undesirable areas [4]. In addition to fat embolism, other life-threatening procedural risks and complications include infection, deep vein thrombosis, pulmonary embolism, complications of anesthesia, and acute blood loss anemia [9]. Less severe, but potential complications include edema, delayed wound healing, seroma, hematoma, scarring, and fat loss or necrosis. Across all complications, the specific risk estimated for mortality of the BBL in 2017 was 1:2351-6241 [10]. Improvement in mortality rates to 1:14,952 was seen across 2019 to 2021 after increased safety measures were instituted [11].

In spite of the associated risks, the number of BBLs performed in the USA increased by 90% from 2015 to 2019, according to the American Society of Plastic Surgeons (ASPS) [12]. With such an increase in popularity, it follows that there is a significant amount of content relating to the BBL on social media. Although becoming safer in recent years, accurate information on social media is crucial to inform the growing patient population of possible risks with the procedure. Accurate online reporting gives patients the knowledge to choose qualified surgeons to perform the procedure safely.

Since its nascence, social media has become a popular medium for sharing content regarding plastic surgery, both for physicians and for patients [13]. Video- and photograph-based social media platforms like Instagram and TikTok have appeal due to the visual nature of plastic surgery. A recent cross-sectional survey of 811 board-certified plastic surgeons reported that 43.9% had a professional Instagram account to distribute information regarding their practice or procedural techniques [14]. Most plastic surgeons use social media to educate public viewers, brand their medical practice, and attract patients [15]. In the current social media landscape, Facebook has been shown to be the most utilized platform for patients and plastic surgeons in the esthetic space [16]. However, because Instagram uses #hashtags as a means for navigating the platform, it is easier for patients to search specific tags to view surgery-specific content [17]. Plastic surgeons also know to include the same hashtags on their posts to boost viewership. This feature has made Instagram come to be used as the ideal platform for plastic surgeons to connect with patients. Thus, it is the preferable channel to study for social media analyses in plastic surgery [17,18].

Due to the widespread use of social media and its prevalence among plastic surgeons and patients, it is clear that social media can play an important role in educating patients on a procedure’s safety and outcomes. However, content regarding BBL on social media has not previously been examined. Therefore, the objective of this study was to analyze public social media content about the BBL procedure on Instagram, with a particular focus on the type of content presented and the qualifications of the author. Considering the risks associated with BBL, it is important to determine how informed the public may be about the procedure from the content available on social media.

## Methods

The picture- and video-sharing social media website Instagram was queried using seven hashtags twice in one month from November to December 2021. These hashtags included: #bbl, #brazilianbuttlift, #bbljourney, #bblrecovery, #bblsafety, #safebbl, and #bblsurgery. Hashtags were selected based off exploratory searches on Instagram and review of popular hashtags. The first 50 relevant posts per hashtag were selected for analysis, where relevance was determined by the post appearing in the “Top” category of the app. Posts were excluded if they were general fan pages, satire pages, advertisements for products or services, in a language other than English, “Reels”, duplicates, or unrelated to BBL. The use of a single social media platform allowed for standardization of data extraction, analysis, and ensured greater ability to draw meaningful conclusions from the data collected. Five independent study team members conducted the search, with at least two members verifying each included post.

**Fig. 1:**
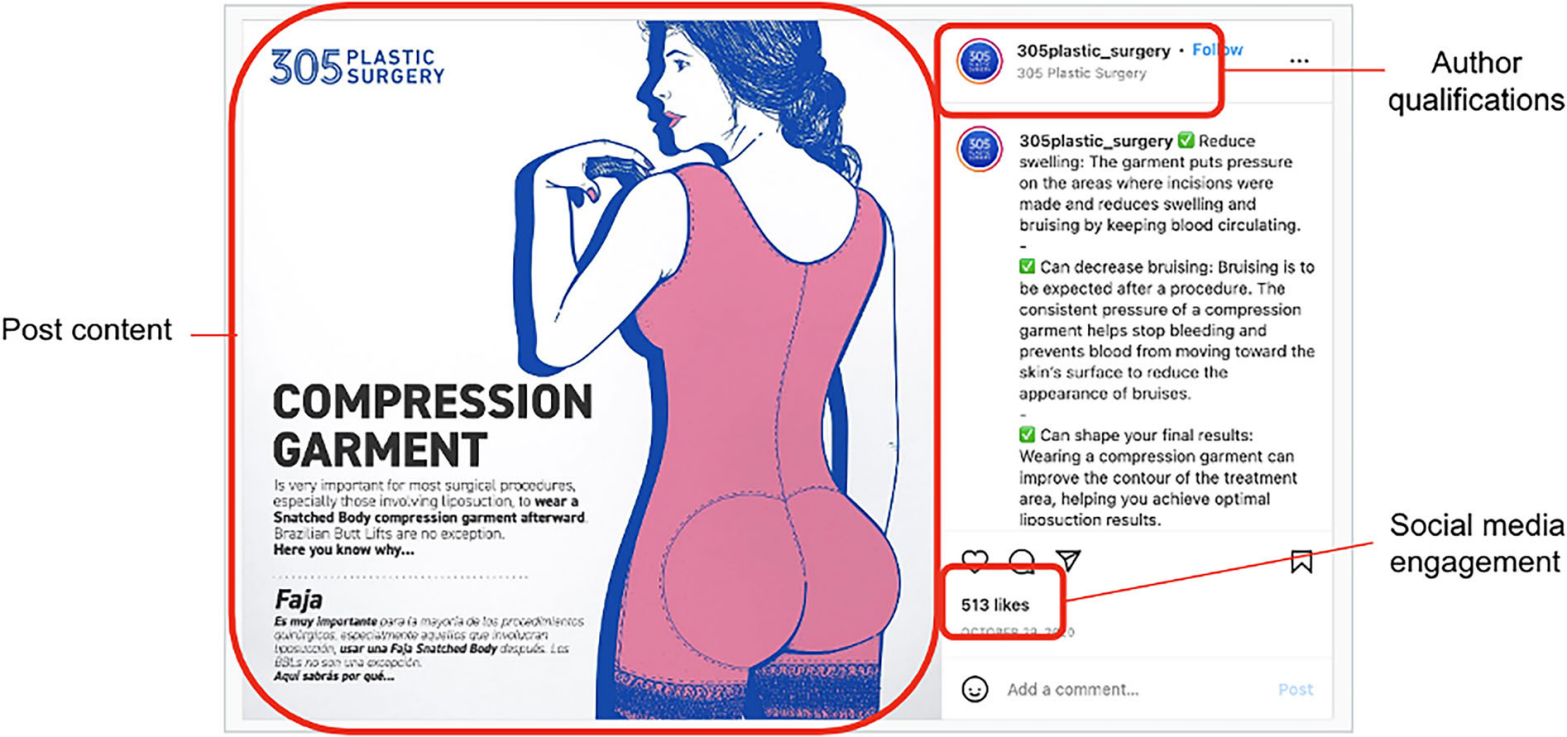
Data extraction technique for a representative BBL-associated Instagram post. From each post identified with this search, extraction included information regarding author identity, author qualifications, post and caption content, and social media engagement (likes and comments). Posts were analyzed by two independent reviewers for consistency

The data extracted from each post included author identity and qualifications, social media engagement (likes and comments), and content (Figure 1). The author identity was categorized into the following groups: physicians, physician groups, patients, non-physician providers, patient coordinators, media (ex. a talk show host or content creator), and academic plastic surgery departments. Among the physicians, board certification status of the author was confirmed via the American Board of Plastic Surgery (ABPS) website so as to include only those surgeons certified by the ABPS. After reviewing all posts, posts were categorized into the following groups: research, safety (including risks of surgery), patient education, surgeon photographs, patient photographs (preoperative, intraoperative, and/or postoperative), satire, and advertisement (which was defined as a post where the viewer was encouraged to make an appointment to discuss surgery). Posts could contain multiple types of content.

Post characteristics were compared with statistical analysis. Descriptive statistics for demographic variables were calculated. Fisher’s exact test and the Chi-square test were used to calculate statistically significant differences in the distributions of separate sets of the categorical groups. T-tests were used to determine significance of distribution of number of likes and comments between the different categorical entities. All data analysis was performed with the Statistical Analysis Software (SAS) Suite (Viya 4) [19].

This study was determined to not qualify as human research by the Northwestern Institutional Review Board, and it was therefore exempt from review.

## Results

A total of 587 posts, with a total of 458,659 “likes” and 9,230 comments, were included in the analysis.

### Author Type/Qualification

The majority (79.0%) of authors were physicians. One-third of all posts were shared by the same five accounts, including three surgeons certified by the ABPS and two physician groups. Of the 128 unique physicians identified by our search, 63.0% were plastic surgeons certified by the ABPS. The rest were cosmetic surgeons (17.0%), plastic surgeons who were not certified by ABPS (13.0%), or physicians whose training or certification could not be verified (8.0%). There were no posts created by any plastic surgery organizations, and only one post created by an academic plastic surgery department.

### General Content

Of the photographic content featuring the Brazilian butt lift, the majority displayed postoperative results (73.0%). Other content, in order of frequency, included preoperative photographs (24.0%), the operating surgeon(s) (16.0%), and intraoperative scenes (13.0%). The states with the greatest representation were Florida (27.0%), Massachusetts (8.0%), California (7.0%), and New York (6.0%).

### Safety and Education Content

While 29.0% of posts included some educational information, only 12.0% mentioned risks and 3.0% referenced research. Plastic surgeons certified by the ABPS contributed 57.0% of the educational posts, but only 33.0% of the posts mentioning risk. The most frequently specified risks were edema (19.0%), fat embolism (18.0%), death (15.0%), and wound healing (15.0%). Other cited risks included bleeding (10.0%), deep vein thrombosis or pulmonary embolism (8.0%), complications of anesthesia (7.0%), seroma (5.0%), and fat loss or necrosis (3.0%). Within educational content, the most commonly discussed topic was surgical technique for the BBL (47.0%). This was followed by postoperative educational content, such as recommendations for postoperative diet and physical activity (35.0%), preoperative information (11.0%), safety (9.0%), appropriate surgical candidate information (3.0%), information regarding choosing a surgeon (3.0%), and anesthesia information (2.0%).

### American Board of Plastic Surgery Certification and Post Content

Posts created by plastic surgeons certified by the ABPS were compared against those made by practitioners not certified by the ABPS. These results are shown in Table 1. ABPS-certified surgeons had a significantly higher number of advertisement posts than practitioners not certified by the ABPS (50.3% compared to 38.5%, p = 0.04). Non-ABPS-certified practitioners had significantly higher numbers of posts related to safety (25.0% compared to 7.4%, p < 0.001) and satire (9.4% compared to 4.0%, p=0.03). Research (defined as a reference to published research) made up 4.6% of ABPS-certified plastic surgeons’ post content, whereas there were no practitioners not certified by the ABPS citing research in their posts; this difference was found to be statistically significant (p=0.02). The most common type of content for both ABPS-certified plastic surgeons and non-ABPS-certified practitioners was postoperative photographs (73.8% versus 67.7%). There was no significant difference in the mean amount of social media engagement (likes and comments) between ABPS-certified surgeons and non-ABPS-certified practitioners.

**Table 1:** Differences in ABPS board-certified plastic surgeons and non-ABPS board-certified practitioners in Instagram content

	Board-certified (%)	Not Board-certified (%)	p
Surgeon photographs	19.8	26.0	0.185
Preoperative photographs	23.5	31.3	0.123
Intraoperative photographs	16.7	16.7	1.000
Postoperative photographs	73.8	67.7	0.244
Research	4.6	0.0	0.028
Patient education	30.6	28.1	0.648
Safety	7.4	25.0	<0.001
Satire	4.0	9.4	0.038
Advertisement	50.3	38.5	0.043

Results reported as percentages.

### Post Content and Social Media Engagement

Social media engagement was then compared across content type (Table 2, Table 3). When post content included research, the mean number of likes (3717.9 ± 4629.8) was significantly higher than non-research posts (737.2 ± 1604.5) (p=0.02). The mean number of comments on research posts was also higher than non-research posts (18.7 ± 29.5, compared to 13.6 ± 26.0), but this difference was not significant (p=0.45). Posts without advertising content garnered significantly more likes than when advertisement posts (1167.0 ± 2114.5 vs 488.0 ± 1488.9, p=0.0001). Posts without advertising also gained more comments (15.1 ± 20.9 vs 12.3 ± 30.8), though this trend was not statistically significant (p=0.27). Patient education content generated significantly more comments than when patient education was not included (18.0 ± 22.6 vs 11.9 ± 27.3, p=0.01). Posts including patient education also received more likes than non-educational posts (1137.2 ± 2315.1 vs 717.9 ± 1634.9) but this finding was not statistically significant (p=0.06).

**Table 2:** Differences in “like” count by Instagram post content

**Content Type**	**Likes if Content Included (mean)**	**Likes if Content Not Included (mean)**	**p-value**
Surgeon photographs	1280.4	726.3	0.057
Preoperative photographs	1056.8	771.7	0.176
Intraoperative photographs	1001.3	812.2	0.441
Postoperative photographs	743.0	1107.6	0.136
Research	3717.9	737.2	0.026
Patient education	1137.2	717.9	0.067
Safety	1379.7	774.5	0.134
Satire	646.1	854.6	0.210
Advertisement	488.0	1167.0	<0.01

Averages represent means. Posts were compared using T-test analysis.

**Table 3:** Differences in comment count by Instagram post content

**Content Type**	**Comments if Content Included (mean)**	**Comments if Content Not Included (mean)**	**p-value**
Surgeon photographs	15.2	13.4	0.516
Preoperative photographs	16.7	12.8	0.133
Intraoperative photographs	15.8	13.4	0.488
Postoperative photographs	13.4	14.7	0.589
Research	18.7	13.6	0.455
Patient education	18.0	12.0	0.020
Safety	13.3	13.8	0.850
Satire	25.4	13.1	0.124
Advertisement	12.3	15.1	0.280

Averages represent means. Posts were compared using T-test analysis.

## Discussion

As social media can serve as a free and accessible venue for plastic surgeons, patients, and other stakeholders to create and share content, it is not surprising that BBL has become a popular topic of discussion. However, our findings demonstrate that the majority of content posted by physicians does not provide valuable information regarding risks and safety of the procedure.

A total of 587 posts were analyzed which had nearly 500,000 likes and 10,000 comments. This high quantity of engagement demonstrated the continued interest in BBL. Of those posts, the location with the highest representation was Florida. A recent study indicated Florida to be one of the states with the greatest number of ABPS member surgeons at 438 [20]. This undoubtedly underestimates the total number of physicians performing BBL, as our data demonstrated that many physicians who are not certified by the ABPS are also posting about BBL. This suggests that plastic surgeons in Florida may be competing among each other to attract patients. Due to the high concentration of providers, there may be pressure to create promotional and advertising-focused content related to their clinical services to attract patients, and less incentive to post education and safety content related to BBL.

Furthermore, of the 128 unique physicians, 63.0% were plastic surgeons verified to be board-certified by the ABPS, while the remaining were practitioners who were not board-certified by the ABPS, “cosmetic” surgeons, or physicians whose training or certification status could not be verified or determined. Other studies of plastic surgery content on social media have found board-certified physicians to be in the minority of content authors, and some authors have found non-board-certified physicians to have a stronger presence [21,22]. Physicians who are board-certified are required to meet certain standards of skill and knowledge, in addition to completing specific training. The importance of board certification by a professional group or society may not be known to the public, so there is a risk that patients may choose a surgeon based on their social media presence alone, without researching the qualifications that the surgeon may have. We hope to encourage the plastic surgery community to produce more educational content on social media regarding the Brazilian butt lift and other popular cosmetic procedures. According to a recent cosmetic surgery patient survey, only 22.1% of patients knew that non-ABPS-certified practitioners can perform esthetic procedures [23]. In addition, it has been demonstrated that it can be challenging to determine whether physicians are board-certified by the ABPS from their websites [24]. Considering the relatively large percent of practitioners who are not certified by the ABPS among our data set, it is possible that patients seeking BBL procedures may choose a practitioner who is not ABPS-certified and may not be adequately informed and trained for the procedure. Since there is little in the way of regulation or oversight for not ABPS-certified surgeons, advertising in this group is easier and likely, as a result, higher in volume. Board-certified plastic surgeons face more regulation, which must be acknowledged in this study.

A minority (13.0%) of posts included discussion of safety risks. Edema, fat embolism, death, and wound healing issues were the risks mentioned the most. As previous studies have explained, fat embolism is the most significant risk of the procedure [25,26]. Thus, it is does not seem that the patient population is being adequately informed of the risks associated with BBL through social media. It is not entirely surprising that few posts discuss risk, especially since many physicians use Instagram as a platform for advertisement. A prior study of social media regarding breast reconstruction and fat grafting on the platform TikTok similarly concluded that content was lacking in the topics of patient education and discussion of risks [27]. Physicians may be concerned that discussing risk could alienate potential patients; however, our results regarding social media engagement suggest that this concern is unfounded. Specifically, safety-informing content resulted in higher numbers of engagement which may correlate with increased visibility and patients’ appreciation of the practice.

Posts including research and patient education had more engagement, while posts with advertisements had less. This finding suggests that the public is interested in learning about educational content relating to BBL. However, research was only featured in a small percentage of posts, and ABPS-certified plastic surgeons provided less content about safety than non-ABPS board-certified practitioners. This highlights an opportunity for ABPS board-certified plastic surgeons to use social media for patient education and sharing research results with their potential patient population without concern that such content would reduce their social media engagement. Plastic surgeons should critically evaluate the type of content they share on social media and consider including more content that educates patients on the BBL procedure, including safety information and risks. Prior work has shown that patients highly value social media posts from surgeons that emphasize high-quality care, rather than those that appear to be working toward monetization in clinics [28]. Our findings surrounding patients’ preference toward safety information and educational content support previous social media analyses.

It is important to highlight the category of authors that was not posting about BBL in our study: plastic surgery organizations and academic departments. This indicates that there is an opportunity for these organizations to join the social media conversation about this procedure and other cosmetic procedures. As opposed to individual plastic surgeons, who have an incentive to make posts that advertise their services, organizations such as the American Society of Plastic Surgeons (ASPS) and the ABPS, and academic departments are uniquely poised to promote new research and educational information about BBL that have the potential to garner significant social media engagement.

### Limitations

This study had multiple limitations, including the cross-sectional study design, the large volume of posts by a few prolific users, and the lack of any formal or validated methodology for evaluating Instagram posts. The search was also performed prior to the implementation of the emergency rules enacted by the Florida Boards of Medicine and Osteopathic Medicine in June 2022 regarding BBL. These rules require the use of ultrasound guidance while performing BBL and limit providers to performing three BBLs per day. These new rules could have affected social media content about BBL. We recognize that Instagram posts may be made more visible using paid engagement, and this may affect generated likes and comments. As these posts cannot be filtered out or labeled, it remains a limitation of social media analyses. In terms of how Instagram or Meta boosts content, the company describes their algorithm for boosting posts as one that relies on “signals” based on how users interact with the app. Boosted content is based on what users press like on, comment on, and how posts are tagged [29]. One limitation of our study is that our group needed to rely on Instagram/Meta’s algorithms during our search to determine the top posts. This drawback applies to all social media analyses though should be acknowledged.

This study highlights the need for better self-regulation by physicians and organizations regarding social media content, and we hope to encourage the plastic surgery community to produce more educational content on social media regarding the Brazilian butt lift and other popular cosmetic procedures. Ways to improve information for patients on social media may take many forms, including but not limited to sharing more peer-reviewed study findings around the BBL, sharing ways that patients can prepare themselves preoperatively to ensure lower risks of complications, and simple acknowledgements of both benefits and complications associated with the procedure. Future studies regarding the intersection of public engagement with esthetic plastic surgery may benefit as well from comparisons between different procedure types and understanding whether any differences exist between gluteal augmentation and other cosmetic surgery procedures.

## Conclusions

Most BBL-related content on Instagram does not provide educational information or promote understanding of the risks of the procedure. There may be a detrimental effect when content is posted by uncertified users that propagate unrealistic expectations for BBL results postoperatively. Posts including research and patient education are associated with more user engagement, suggesting that these are topics of interest that can be disseminated through social media. Social media remains an underutilized mechanism for patient education, and plastic surgery organizations and surgeons should consider using social media to disseminate information on the risks and indications of BBL and other popular cosmetic procedures.
